# Focusing on depressive symptoms in patients to mitigate the risk of pain: a nationwide, prospective cohort study

**DOI:** 10.1186/s12889-026-28359-0

**Published:** 2026-07-03

**Authors:** Yao-Wen Liu, Yi Sun, Yu-Lin Lei, Bin-Ru Di, Xiao-Ding Men, Jing-Zhou Zhu, Kai-Xiang He, Jian-Mei Wang, Lin Peng, Li Xiang, Yu-Hao Luo

**Affiliations:** 1 Department of Oncology, The Affiliated Hospital of Southwest Medical University, Luzhou, 646000 China; 2 Department of plastic&burn surgery, The Affiliated Hospital of Southwest Medical University, Luzhou, 646000 China; 3 Department of Pathology, The Affiliated Hospital of Southwest Medical University, Luzhou, 646000 China; 4 Department of Bone and Joint, The Affiliated Hospital of Southwest Medical University, Luzhou, 646000 China

**Keywords:** Depressive, pain, CESD scores, Cohort study, China Health and Retirement Longitudinal Study

## Abstract

**Background:**

Depressive symptoms and pain frequently coexist and contribute substantially to individual and public health burdens. Although previous studies have demonstrated a bidirectional relationship between depressive symptoms and pain, several important knowledge gaps remain. Most investigations have focused on overall pain burden or specific pain conditions, limiting understanding of whether depressive symptoms are associated with the risk of pain across different anatomical sites. In addition, the dose–response relationship between depressive symptom severity and subsequent pain risk remains poorly characterized, particularly in nationally representative Chinese populations.

**Methods:**

The China Health and Retirement Longitudinal Study (CHARLS), a national, multi-center, prospective cohort study initiated in 2011, enrolled 6,772 participants for the final analysis, based on specific inclusion and exclusion criteria. Depressive symptoms was defined as a Center for Epidemiologic Studies Short Depression (CESD) score ≥ 10. To investigate potential associations between depressive symptoms, CESD scores, and 15 distinct types of pain, Cox proportional hazards models were employed, with restricted cubic splines (RCS) applied to examine non-linear relationships. Lastly, subgroup analyses were performed to substantiate the reliability of the findings further.

**Results:**

In the fully adjusted model, individuals with depressive symptoms exhibited a significantly higher risk of experiencing pain in specific body regions compared to their non-depressed counterparts. Furthermore, for every 1 standard deviation increase in CESD score, the risk of pain in certain body areas rose by 4%-7%. Moreover, CESD scores displayed a distinct S-shaped non-linear relationship with ten types of pain, including headache and shoulder ache. Additionally, in the majority of subgroups, both depressive symptoms and CESD scores were positively correlated with pain risk, aligning with the overall findings of the study.

**Conclusions:**

In the Chinese population, depressive symptoms and CESD scores represent significant potential risk factors for pain.

**Supplementary Information:**

The online version contains supplementary material available at https://doi.org/10.1186/s12889-026-28359-0.

## Introduction

Depression is a heterogeneous psychiatric disorder marked by persistent sadness, a pervasive sense of hopelessness, and a diminished interest in activities that were once deemed pleasurable. In more severe instances, depression can lead to significant fluctuations in weight, insomnia, cognitive impairments, and recurrent thoughts of death or suicide [1]. Furthermore, depression is a chronic and debilitating condition that substantially impairs social, occupational, and other critical aspects of life, significantly reducing overall quality of life [2, 3]. Depression is the most prevalent of all mental disorders, affecting a substantial portion of the population worldwide. A large-scale epidemiological study revealed that the prevalence of depression stands at 22.1% in the United States, 34.8% in Japan, 34.6% in France, and 42.0% in China [4]. According to the World Health Organization’s 2021 data, approximately 280 million individuals worldwide are affected by depression, rendering it both the leading cause of disability and a significant contributor to the global disease burden [5]. Consequently, relevant organizations have implemented the Center for Epidemiologic Studies Short Depression (CESD) scale to facilitate more effective screening of depression prevalence in large populations [6].

Pain is a pervasive adaptive sensation that serves a protective function for individuals; however, it can also exert detrimental effects on individual functionality, as well as on social and mental health [7]. According to the latest guidelines from the International Association for the Study of Pain (IASP), pain is defined as an unpleasant sensory and emotional experience linked to actual or potential tissue damage, or other similar factors [8]. In the existing literature, researchers commonly classify pain based on its location, such as headache, back ache, chest ache, and arm ache [9]. Although pain in various regions is frequently accompanied by potential tissue damage, several studies have highlighted the close relationship between pain and emotional and mental states. The biopsychosocial model of pain conceptualizes bodily symptoms as the outcome of dynamic interactions among biological, psychological, and social factors [10, 11].

Depressive symptoms, as a mental illness predominantly affecting an individual’s emotional state, undoubtedly exerts a profound impact on pain [12]. Epidemiological studies have demonstrated that individuals with severe depressive symptomsare three times more likely to experience non-neuropathic pain and six times more likely to experience neuropathic pain, compared to the general population [13]. Unfortunately, in clinical practice, healthcare professionals frequently fail to give adequate attention to the pain that may accompany depressive symptoms [14]. Although accumulating evidence suggests a close and potentially bidirectional relationship between depressive symptoms and pain, several important gaps in the current literature remain [15, 16]. First, most prospective studies have been conducted in Western populations, whereas nationally representative longitudinal evidence from China is still limited. Second, previous research has primarily focused on overall pain burden or specific pain conditions, with relatively few studies comprehensively examining pain across multiple anatomical sites. Third, the nature of the association between depressive symptom severity and subsequent pain risk remains poorly understood, particularly regarding whether this relationship is linear or non-linear. To address these gaps, we used data from a nationwide prospective cohort to investigate the association between depressive symptoms and the risk of incident pain at multiple anatomical sites and to characterize potential dose–response relationships using restricted cubic spline analyses.

## Method

### Study design and population

The China Health and Retirement Longitudinal Study (CHARLS) is a nationwide, prospective cohort study conducted in China, designed to gather high-quality epidemiological data representing households and individuals aged 45 and above, to analyze issues related to population aging and fostering interdisciplinary research on aging. The national baseline survey of CHARLS was initiated in 2011, encompassing 150 county-level units and 450 township-level units, with a total of 17,708 participants from 10,257 households. Follow-up surveys were subsequently conducted by trained professionals in 2013, 2015, 2018, and 2020 [17].

**Fig. 1:**
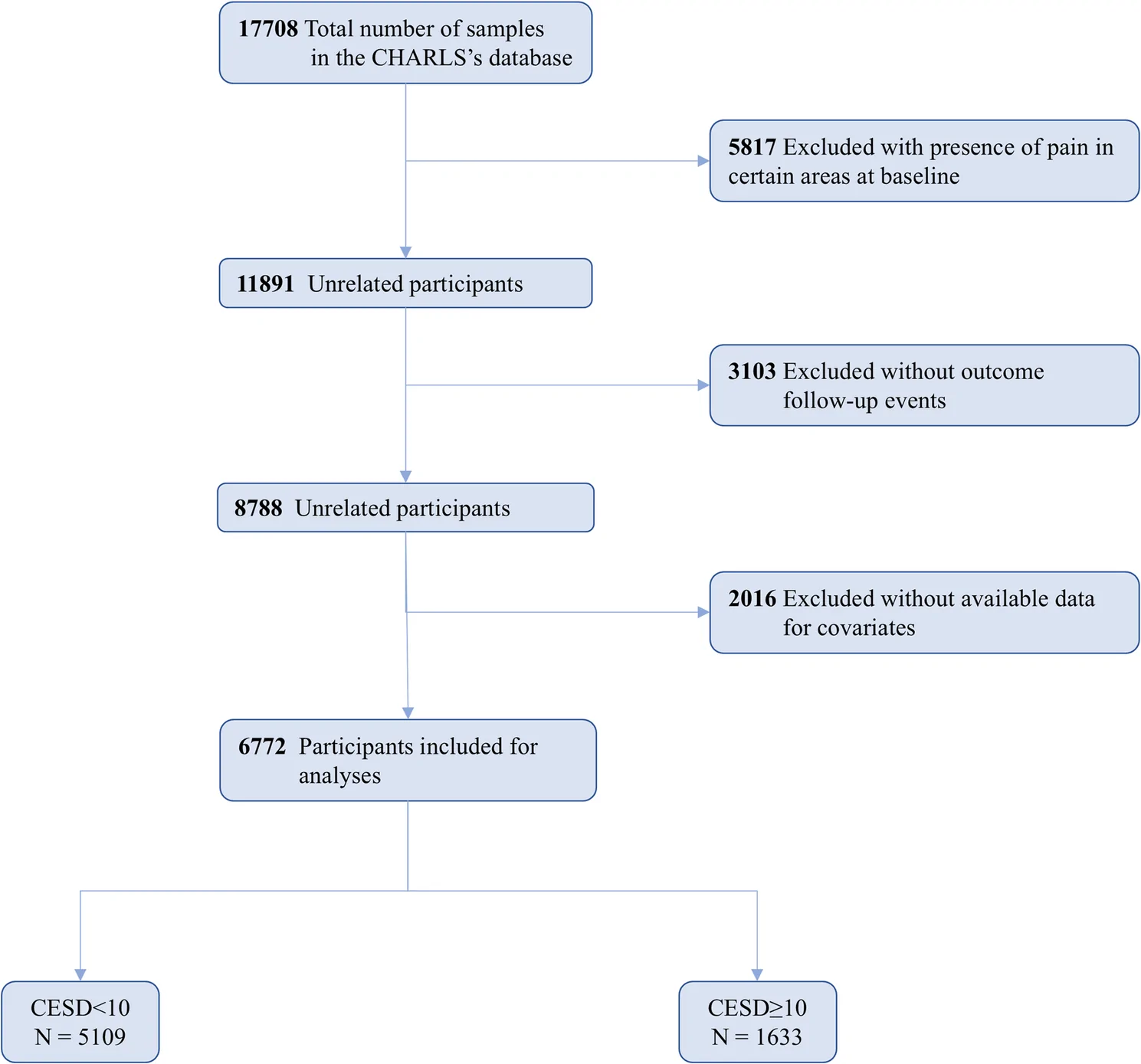
Flowchart for the Selection of the Analyzed Study Sample From the CHARLS’s database

In this study, 5,817 participants were excluded due to pre-existing pain at baseline, 3,103 participants were excluded due to missing follow-up data, and 2,016 participants were excluded due to incomplete covariate data. Ultimately, a total of 6,772 participants were included in the final analysis (see Fig. 1).

### Data collection

By the data collection guidelines of CHARLS, demographic, physical, and laboratory examination data for all participants were collected by community workers who had received professional training. Blood pressure was measured after participants rested in a seated position for five minutes, with the measurement being repeated three times to obtain an average value. Physical measurements, including weight, height, and waist circumference (WC), were recorded while participants wore light clothing. Additionally, all blood samples were collected by trained professionals after participants fasted overnight, stored at -20 °C, and subsequently transported to Beijing for further analysis, following standardized procedures. Biochemical indicators included serum creatinine (Cr), glycated hemoglobin A1c (HbA1c), blood lipid levels, and other relevant markers. Smoking history was assessed with the question: “Have you ever chewed tobacco, smoked a pipe, smoked cigarettes, or used cigars?” The possible responses were: (1) Yes; (2) No; or (3) Quit. Alcohol consumption history was assessed with the question: “In the past year, have you consumed alcoholic beverages, such as beer, wine, or spirits?” The possible responses were: (1) More than once a month; (2) Less than once a month; or (3) Do not drink alcohol [18].

### Assessment of CESD score and depressive

The CHARLS study primarily utilizes the CESD-10 scale to screen participants for depressive symptoms [19]. Respondents were asked to report the number of days in the past week that they experienced each symptom. Each item was rated using a 4-point Likert scale: 0 (Rarely or never; less than 1 day), 1 (Some of the time; 1–2 days), 2 (Most or a moderate amount of the time; 3–4 days), or 3 (All or almost all of the time; 5–7 days). The total score ranges from 0 to 30, with lower scores reflecting less severe depressive symptoms. Additionally, a CESD score of ≥ 10 was defined as indicative of depressive symptoms [20].

### Assessment of body pain

In all five waves of the CHARLS survey, physical pain in participants was assessed through personal interviews and questionnaires. The questionnaire contained a multiple-choice question: “Which areas of your body are experiencing pain? Please list all the body parts where you are currently feeling pain. “Participants could select from 15 body parts, including headache, shoulder ache, arm ache, wrist ache, finger ache, chest ache, stomach ache, back ache, lower back ache, hip ache, leg ache, knee ache, ankle ache, toe ache, and neck ache [21]。.

### Ascertainment of covariates

By prior studies, we incorporated a range of potential confounding variables into the baseline statistical analysis [22]. The covariates included age, gender (classified as “male” or “female”), marital status (categorized as “unmarried or divorced” and “married”), smoking status (“current smoker” or “non-smoker”), alcohol consumption (“yes” or “no”), and medical history (including diabetes, hypertension, stroke, and heart disease, all classified as “yes” or “no”). Furthermore, we adjusted for a variety of clinical indicators, including systolic blood pressure (SBP), diastolic blood pressure (DBP), body mass index (BMI), waist circumference (WC), fasting blood glucose, total cholesterol (TC), triglycerides (TG), creatinine (Cr), high-density lipoprotein cholesterol (HDL-c), low-density lipoprotein cholesterol (LDL-c), and glycated hemoglobin (HbA1c).

### Statistical analysis

The baseline characteristics of the participants were thoroughly described using appropriate statistical techniques tailored to the data types. Continuous variables were presented as mean ± standard deviation (SD), while categorical variables were expressed as counts (percentages). For normally distributed data, analysis of variance (ANOVA) was employed to compare group differences, whereas Kruskal-Wallis tests were applied for non-normally distributed data. Chi-square tests were conducted to compare differences in categorical variables between groups. Subsequently, Cox proportional hazards regression models were employed to assess the relationship between depressive symptoms, CESD scores, and the 15 types of body pain. A fully adjusted model was developed to estimate the hazard ratios (HR) and 95% confidence intervals (CIs). The fully adjusted model accounted for age, gender, WC, BMI, DBP, SBP, LDL-C, TG, Cr, HbA1c, marital status, smoking history, alcohol consumption history, and medical history (including diabetes, hypertension, stroke, and heart disease). Furthermore, based on the fully adjusted model, four-knot restricted cubic splines were employed to assess the potential non-linear relationship between CESD scores and the risk of 15 types of body pain, with four knots selected according to established methodological recommendations to provide adequate flexibility for modeling non-linear associations while minimizing the risk of overfitting [23, 24]. Subgroup analyses were subsequently conducted based on age (< 60, ≥ 60), gender (male, female), smoking history (yes, no), and alcohol consumption history (yes, no) to examine the interaction between CESD scores and various subgroup factors. A non-significant interaction P value indicated consistency across strata, whereas a significant P value suggested potential subgroup-specific effects.

Statistical analyses were performed using SPSS version 26.0 and R version 4.3.3. A two-sided P value of less than 0.05 was deemed statistically significant.

## Result

### Baseline characteristics

Table 1 presents the baseline characteristics of participants stratified by depressive symptoms status. This study involved 6,772 participants (mean age: 57.52 ± 9.07 years), with 3,345 males (49.39%). Compared to non-depressed participants, individuals with depressive symptoms were older and exhibited higher levels of LDL-C, HDL-C, TC, and HbA1c. Additionally, a greater proportion of participants with depressive symptoms had a history of hypertension, stroke, and cardiovascular diseases. In contrast, participants with depressive symptoms had lower levels of WC, DBP, SBP, TG, and Cr, as well as a lower proportion who were married, smokers, or drinkers (All *P* < 0.05).

**Table 1:** Baseline characteristics of participants by history of depression

Variables	Tatal	Non-depressed	Depressed	*P*
N	6772	5109	1663	
Age, years	57.52 ± 9.07	57.30 ± 9.06	58.20 ± 9.08	< 0.001
Male (%)	3345(49.39)	2695(52.75)	650(39.09)	< 0.001
BMI, kg/m^2^	24.07 ± 3.42	24.35 ± 3.86	23.21 ± 3.94	0.11
WC, cm	84.47 ± 12.17	84.70 ± 12.33	83.78 ± 11.65	0.01
DBP, mmHg	75.29 ± 11.98	75.66 ± 11.84	74.16 ± 12.34	< 0.001
SBP, mmHg	128.31 ± 20.45	128.68 ± 20.18	127.14 ± 21.23	0.01
LDL-c, mg/dl	117.03 ± 35.35	116.06 ± 35.26	119.93 ± 35.47	< 0.001
HDL-c, mg/dl	50.79 ± 15.14	50.34 ± 14.98	52.13 ± 15.56	< 0.001
TC, mg/dl	193.76 ± 39.61	192.71 ± 39.57	196.1 ± 39.91	< 0.001
TG, mg/dl	134.27 ± 109.59	134.82 ± 109.02	132.64 ± 111.32	0.54
Cr, mg/dl	0.77 ± 0.18	0.78 ± 0.18	0.75 ± 0.19	< 0.001
FBG, mg/dl	109.19 ± 33.22	109.01 ± 31.82	109.70 ± 37.13	0.53
HbA1c, %	5.24 ± 0.75	5.22 ± 0.73	5.29 ± 0.82	< 0.001
Married	6125(90.45)	4705(92.09)	1420(85.39)	< 0.001
Smoking	2657(39.24)	2077(40.65)	580(34.88)	< 0.001
Alcohol consumption	2672(39.46)	2077(40.65)	595(35.77)	< 0.001
Diabetes	324(4.78)	240(4.70)	84(5.05)	0.57
Hypertensive	1515(22.37)	1100(21.53)	415(24.95)	< 0.001
Stroke	101(1.49)	65(1.27)	36(2.16)	0.01
Cardiology	555(8.20)	386(7.56)	169(10.16)	< 0.001

Data are summarized as number (percentage), mean ± standard deviation

*BMI* body mass index, *WC* waist circumference, *DBP* diastolic blood pressure, *SBP* systolic blood pressure, *LDL-c *low-density lipoprotein cholesterol, *HDL-c* high-density lipoprotein cholesterol, *TC* total cholesterol, *TG* triglyceride, *Cr* creatinine, *FBG* fasting blood glucose, *HbA1c* glycated haemoglobin

### Association of depressive symptoms and CESD score with pain

**Fig. 2:**
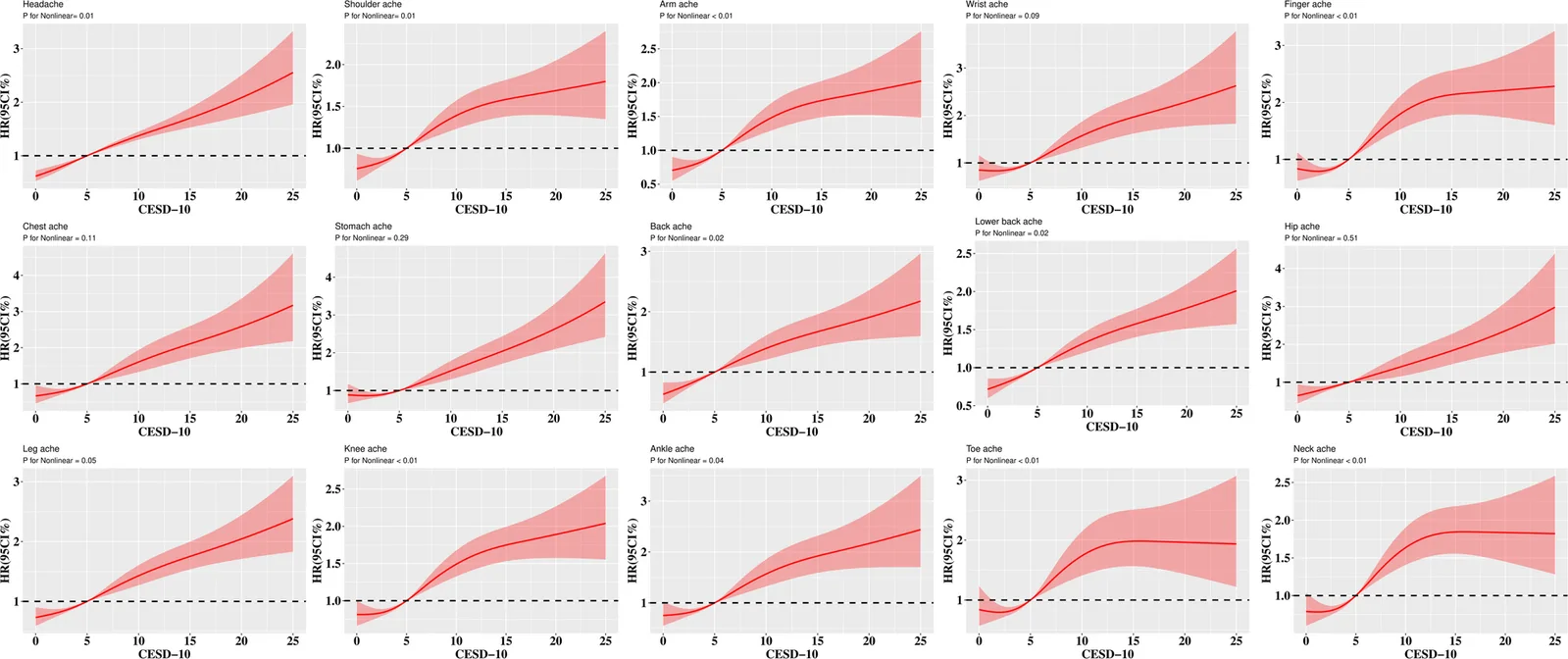
Results of RCS analysis of the association between CESD score and pain. Adjusted for age, sex, WC, BMI, DBP, SBP, HDL-c, LDL-c, TC, TG, Cr, HbA1c, marital status, smoking, alcohol consumption status, history of diabetes, history of hypertension, history of stroke, and history of heart disease. CESD, Center for Epidemiological Studies Depression Scale; HDL-c, glucose index; BMI, body mass index; WC, waist circumference; DBP, diastolic blood pressure; SBP, systolic blood pressure; LDL-c, low-density lipoprotein cholesterol; TG, triglyceride; Cr, creatinine

Table 2 illustrates the findings from the Cox proportional hazards regression model. In the fully adjusted model, compared to non-depressed individuals, those with depressive symptoms were at a higher risk of experiencing pain in multiple body areas. The highest HR was observed for chest pain (HR: 2.02, 95% CI: 1.71–2.39), while the lowest HR was for shoulder pain (HR: 1.53, 95% CI: 1.37–1.72) (All *P* < 0.05). Additionally, for each 1 standard deviation increase in the CESD score, the risk of experiencing pain in various body areas increased by 4%-7% (All *P* < 0.05). Figure 2 illustrates the results from the RCS analysis. In the fully adjusted model, the CESD score displayed a significant S-shaped non-linear association with headache, shoulder ache, arm ache, finger ache, back ache, lower back ache, knee ache, ankle ache, toe ache, and neck ache(P for nonlinear < 0.05).

**Table 2:** Hazard ratios and 95% CIs for the association of depression and CESD scores and pain at various sites throughout the body

Variables	Non-depressed	Depressed	Per SD
Headache			
Event(%)	940(18.40)	723(43.48)	
Fully adjusted model	Reference	1.76(1.57,1.97)	1.06(1.05,1.07)
Shoulder ache			
Event(%)	935(18.30)	728(43.78)	
Fully adjusted model	Reference	1.53(1.37,1.72)	1.04(1.03,1.05)
Arm ache			
Event(%)	1060(20.75)	603(36.26)	
Fully adjusted model	Reference	1.63(1.44,1.85)	1.05(1.04,1.06)
Wrist ache			
Event(%)	1231(24.09)	450(27.06)	
Fully adjusted model	Reference	1.74(1.49,2.02)	1.06(1.04,1.07)
Finger ache			
Event(%)	1179(23.08)	484(29.10)	
Fully adjusted model	Reference	1.87(1.63,2.16)	1.06(1.05,1.07)
Chest ache			
Event(%)	1292(25.29)	371(22.31)	
Fully adjusted model	Reference	2.02(1.71,2.39)	1.07(1.06,1.09)
Stomach ache			
Event(%)	1179(23.08)	484(29.10)	
Fully adjusted model	Reference	1.91(1.66,2.21)	1.06(1.05,1.08)
Back ache			
Event(%)	1091(21.35)	572(34.40)	
Fully adjusted model	Reference	1.73(1.52,1.97)	1.05(1.04,1.06)
Lower back ache			
Event(%)	732(14.52)	931(55.98)	
Fully adjusted model	Reference	1.56(1.42,1.72)	1.05(1.04,1.05)
Hip ache			
Event(%)	1335(26.13)	728(43.78)	
Fully adjusted model	Reference	1.74(1.47,2.07)	1.06(1.05,1.08)
Leg ache			
Event(%)	879(17.20)	784(47.14)	
Fully adjusted model	Reference	1.65(1.48,1.84)	1.05(1.04,1.06)
Knee ache			
Event(%)	889(17.40)	774(46.54)	
Fully adjusted model	Reference	1.56(1.40,1.74)	1.05(1.04,1.06)
Ankle ache			
Event(%)	1239(24.25)	424(25.50)	
Fully adjusted model	Reference	1.75(1.51,2.04)	1.06(1.05,1.07)
Toe ache			
Event(%)	1373(26.87)	290(17.44)	
Fully adjusted model	Reference	1.70(1.42,2.05)	1.05(1.04,1.07)
Neck ache			
Event(%)	1163(22.76)	500(30.66)	
Fully adjusted model	Reference	1.62(1.42,1.86)	1.05(1.04,1.06)

Fully adjusted model: adjusted for age, sex, WC, BMI, DBP, SBP, HDL-c, LDL-c, TC, TG, Cr, HbA1c, marital status, smoking, alcohol consumption status, history of diabetes, history of hypertension, history of stroke, and history of heart disease

*CESD* Center for Epidemiological Studies Depression Scale, *HDL-c* glucose index, *BMI* body mass index, *WC* waist circumference, *DBP* diastolic blood pressure, *SBP* systolic blood pressure, *LDL-c* low-density lipoprotein cholesterol, *TG* triglyceride, *Cr* creatinine

### Subgroup analyses

To further assess the robustness of our findings, we conducted subgroup analyses stratified by age, sex, smoking status, and alcohol consumption status (Tables S1–S4). Overall, depressive symptoms and CES-D scores remained positively associated with pain risk across most 

subgroups, consistent with the primary analyses. Importantly, formal interaction testing identified a significant interaction between alcohol consumption status and depressive symptoms/CES-D scores for headache risk (P for interaction < 0.05), suggesting that the strength of this association may vary according to drinking status. Within individual strata, significant associations were observed for headache and wrist ache among participants aged ≥ 60 years, headache and neck ache among men, headache and chest ache among current smokers, and headache among current drinkers. However, these subgroup-specific hazard ratios reflect associations within each stratum and should not be interpreted as direct comparisons between strata.

## Discussion

Consistent with previous studies reporting reciprocal associations between depressive symptoms and pain, our findings extend the existing evidence by demonstrating a dose-dependent association between depressive symptom severity and the risk of incident pain across multiple anatomical sites in a nationally representative Chinese population. Furthermore, restricted cubic spline analyses enabled a detailed characterization of the dose–response relationship, revealing the shape of the association and providing epidemiological insights beyond those afforded by conventional linear models.

Although pain serves as one of the body’s most valuable alarm systems, it frequently evokes negative emotional experiences, leading to distress [25]. The latest International Classification of Diseases (ICD) guidelines categorize pain into seven distinct types, including cancer pain, neuropathic pain, and post-traumatic pain [26]. However, due to the limitations of the CHARLS dataset, this study primarily focused on fifteen types of pain, categorized by body region, with back pain being the most prevalent [27]. Overall, a study of the general population revealed that the prevalence of pain in the elderly is as high as 55% in those aged over 60, increasing to 62% in those over 75 [28]. However, due to variations in the study populations and methodologies, the prevalence of pain in the general population exhibits substantial heterogeneity [29, 30]. Regardless of region, the distress caused by pain is undeniable.

Trauma, disease, emotions, and unhealthy lifestyle habits are currently considered common risk factors for pain [31]. The comorbidity of depressive symptoms and pain has long been a subject of research interest [32]. Previous studies have shown that depressive symptoms and pain can simultaneously activate and/or alter human anatomical structures, including the insular cortex, prefrontal cortex, anterior cingulate cortex, amygdala, and hippocampus, and activate shared neural circuits (such as the hypothalamic-pituitary-adrenal axis, limbic structures, and ascending and descending pain pathways), as well as release common neurochemicals (such as monoamines, cytokines, and neurotrophic factors) [33]. In animal models, widely accepted depressive symptoms models use pain as a stressor to induce similar symptoms [34]. This suggests that depressive symptoms and pain share fundamental pathophysiological characteristics, with each serving as a risk factor for the other. Notably, our study also identified a potential association between CESD scores and pain, suggesting that even in non-depressed individuals, it is crucial to lower CESD scores. This may be because non-depressed individuals can still experience negative emotions, and clinical efforts should focus on controlling these emotions to prevent pain resulting from them.

Our findings are broadly consistent with previous longitudinal studies demonstrating that depressive symptoms increase the risk of subsequent pain [35, 36]. However, the present study extends the existing literature by simultaneously evaluating multiple pain sites and characterizing the dose–response relationship between depressive symptom severity and incident pain in a nationally representative Chinese cohort. Several biological and behavioral mechanisms may underlie these associations. Depressive symptoms and pain share common neurobiological pathways, including alterations in serotonergic and noradrenergic signaling, dysregulation of the hypothalamic–pituitary–adrenal axis, neuroinflammatory activation, and central sensitization [37, 38]. In addition, depressive symptoms may contribute to behavioral changes, such as reduced physical activity, poor sleep quality, and heightened attention to somatic symptoms, all of which may increase vulnerability to pain [39]. From a clinical and public health perspective, these findings highlight the importance of early identification and management of depressive symptoms as a potential strategy to reduce the future burden of pain and improve overall health outcomes. Nevertheless, several limitations should be considered when interpreting our findings. In particular, depressive symptoms were assessed using the CES-D, a screening instrument rather than a diagnostic tool, which may have resulted in some degree of misclassification and self-report bias.

In the subgroup analyses, positive associations between depressive symptoms and the risks of headache and neck pain were observed among male participants. However, these findings should be interpreted cautiously, as subgroup-specific estimates reflect associations within strata and do not constitute direct comparisons between men and women. Although previous studies have documented important sex differences in the prevalence, clinical manifestations, and outcomes of depressive symptoms, the extent to which sex modifies the association between depressive symptoms and pain remains unclear. Potential explanations include sex-related differences in neuroendocrine regulation, pain processing, psychosocial factors, and health-seeking behaviors, all of which may influence the perception, reporting, and experience of pain. Nevertheless, the mechanisms underlying potential sex-related heterogeneity in the association between depressive symptoms and pain remain incompletely understood and warrant further investigation [40].

Positive associations were also observed among current smokers and drinkers. Smoking, alcohol consumption, depressive symptoms, and pain have been linked through several shared biological and behavioral pathways, including chronic inflammation, dysregulation of stress-response systems, and unhealthy lifestyle behaviors. For example, smoking has been associated with greater pain burden, increased pain-related disability, and more frequent use of analgesic medications. These shared mechanisms may partially explain the observed associations in these subgroups. However, because the present study was not designed to investigate the underlying mechanisms or establish differential effects across subgroups, these findings should be considered exploratory and require confirmation in future studies [41].

It should be noted that the pain outcomes examined in this study represent anatomically and clinically heterogeneous conditions with potentially distinct underlying etiologies. Accordingly, the observed associations should not be interpreted as evidence that depressive symptoms influence all pain sites through a single biological mechanism [42]. Rather, depressive symptoms may increase susceptibility to pain across multiple anatomical locations through shared processes, including altered central pain modulation, heightened pain sensitivity, dysregulation of stress-response systems, and increased somatic symptom perception. However, the present study was not designed to identify site-specific biological mechanisms. Further research is therefore needed to determine whether distinct pathophysiological pathways contribute to the associations observed across different pain locations.

### Strengths and limitations

This study offers several notable advantages. First, CHARLS demonstrates a high degree of population representativeness, making it an accurate reflection of the middle-aged and elderly population in China. Second, CHARLS benefits from an extended follow-up period and large sample size, offering a stable framework for this study that reduces the inherent biases of cross-sectional designs, thereby enhancing the reliability of the results. Third, although an association between depressive symptoms and pain has been reported previously, the present study extends the existing literature in several important ways. First, our findings provide prospective evidence from a nationally representative Chinese population, thereby broadening the generalizability of previous observations derived primarily from Western cohorts. Second, we simultaneously evaluated multiple anatomical pain sites and characterized the dose–response relationship between depressive symptom severity and future pain risk using restricted cubic spline analyses. These findings suggest that the effect of depressive symptoms on pain risk is graded rather than simply dichotomous, providing a more nuanced understanding of the relationship between depressive symptoms and pain. Finally, the use of the fully adjusted Cox model, RCS, and subgroup analyses strengthens the statistical power, ensuring the robustness and consistency of the results.

Nevertheless, this study is not without its limitations. First, despite the inclusion of numerous potential confounding variables in the fully adjusted model, unmeasured factors, such as dietary patterns, racial differences, medication use, and lifestyle factors, may still introduce bias into the results. Second, given that the participants in this study are predominantly from China, caution is warranted when generalizing the findings to other populations. Third, the inherent limitations of observational studies prevent the establishment of a clear causal relationship. Fourth, recall bias remains an inherent concern in survey-based studies. Fifth, although the CES-D has been extensively validated for assessing depressive symptoms in epidemiological studies, it is a screening instrument rather than a diagnostic tool and therefore cannot establish a formal clinical diagnosis of depressive disorder. Consequently, some degree of symptom misclassification is inevitable. Furthermore, pain was assessed only at discrete follow-up visits, precluding determination of the exact timing of pain onset between survey waves. In addition, because pain is a dynamic condition that may remit and recur over time, analyses based on the first reported pain event during follow-up may not fully capture the complex temporal patterns of pain onset, remission, and recurrence. Consequently, the observed associations should be interpreted in the context of these methodological constraints. Future studies incorporating repeated-measures designs and analytical approaches tailored to interval-censored or recurrent events are warranted to provide a more comprehensive characterization of these temporal relationships.

## Conclusions

In conclusion, our findings confirm and extend existing evidence linking depressive symptoms to subsequent pain by demonstrating dose–response relationships across multiple anatomical pain sites in a nationally representative Chinese population. These results underscore the potential importance of early identification and management of depressive symptoms as a strategy to reduce the future burden of pain and improve population health.

We thank the CHARLS team for providing the data and guidelines for using the dataset. We thank all the volunteers and staff who participated in this study.

## Supplementary Information

Below is the link to the electronic supplementary material.

Supplementary Material 1

## Data Availability

The datasets generated and/or analyzed during the current study are not publicly available but are available from the corresponding author on reasonable request.

## Abbreviations

CESD: Center for Epidemiologic Studies Short Depression
IASP: International Association for the Study of Pain
CHARLS: China Health and Retirement Longitudinal Study
WC: waist circumference
Cr: creatinine
SBP: systolic blood pressure
DBP: diastolic blood pressure
BMI: body mass index
TC: total cholesterol
TG: triglycerides
HDL-c: high-density lipoprotein cholesterol
LDL-c: low-density lipoprotein cholesterol
HbA1c: glycated hemoglobin
SD: standard deviation
HR: hazard ratios
CIs: confidence intervals
ICD: International Classification of Diseases
