# Supplementary material for: Focusing on depressive symptoms in patients to mitigate the risk of pain: a nationwide, prospective cohort study
Source: BMC Public Health. 2026 Jul 3;26:2836. doi: 10.1186/s12889-026-28359-0 (PMC13643971; doi:10.1186/s12889-026-28359-0)
Supplement: Supplementary file 1 [file 12889_2026_28359_MOESM1_ESM.docx]

**Supplementary material**

| **Table S1 Hazard ratios and 95% CIs for the association of depression and CESD scores and pain at various sites throughout the body were calculated using age as a subgroup** | | | | | |
| --- | --- | --- | --- | --- | --- |
| **Variables** | **Non-depressed** | **Depressed** | **P for interaction(Depressed)** | **Per SD** | **P for interaction(CESD)** |
| **Headache** | | | | | |
| <60 | Reference | 1.55(1.33,1.80) | 0.03 | 1.05(1.04,1.06) | 0.13 |
| ≥60 | Reference | 2.10(1.75,2.51) |  | 1.07(1.05,1.08) |  |
| **Shoulder ache** | | | | | |
| <60 | Reference | 1.45(1.26,1.68) | 0.30 | 1.04(1.03,1.05) | 0.34 |
| ≥60 | Reference | 1.65(1.37,1.97) |  | 1.05(1.03,1.06) |  |
| **Arm ache** | | | | | |
| <60 | Reference | 1.54(1.31,1.81) | 0.28 | 1.05(1.03,1.06) | 0.17 |
| ≥60 | Reference | 1.77(1.45,2.15) |  | 1.06(1.04,1.07) |  |
| **Wrist ache** | | | | | |
| <60 | Reference | 1.50(1.22,1.83) | 0.01 | 1.05(1.03,1.07) | 0.32 |
| ≥60 | Reference | 2.16(1.70,2.76) |  | 1.07(1.05,1.09) |  |
| **Finger ache** | | | | | |
| <60 | Reference | 1.82(1.52,2.19) | 0.46 | 1.0691.05,1.08) | 0.97 |
| ≥60 | Reference | 1.94(1.55,2.43) |  | 1.06(1.04,1.08) |  |
| **Chest ache** | | | | | |
| <60 | Reference | 1.75(1.40,2.18) | 0.08 | 1.07(1.05,1.09) | 0.60 |
| ≥60 | Reference | 2.50(1.94,3.24) |  | 1.08(1.06,1.10) |  |
| **Stomach ache** | | | | | |
| <60 | Reference | 1.91(1.59,2.30) | 0.71 | 1.06(1.05,1.08) | 0.85 |
| ≥60 | Reference | 1.90(1.52,2.38) |  | 1.06(1.04,1.08) |  |
| **Back ache** | | | | | |
| <60 | Reference | 1.61(1.35,1.91) | 0.62 | 1.05(1.04,1.07) | 0.89 |
| ≥60 | Reference | 1.93(1.57,2.35) |  | 1.06(1.04,1.08) |  |
| **Lower back ache** | | | | | |
| <60 | Reference | 1.56(1.37,1.77) | 0.37 | 1.05(1.04,1.06) | 0.50 |
| ≥60 | Reference | 1.57(1.35,1.83) |  | 1.05(1.04,1.06) |  |
| **Hip ache** | | | | | |
| <60 | Reference | 1.44(1.14,1.82) | 0.09 | 1.05(1.03,1.07) | 0.21 |
| ≥60 | Reference | 2.22(1.72,2.90) |  | 1.08(1.06,1.10) |  |
| **Leg ache** | | | | | |
| <60 | Reference | 1.57(1.36,1.82) | 0.36 | 1.05(1.04,1.06) | 0.97 |
| ≥60 | Reference | 1.73(1.48,2.03) |  | 1.05(1.04,1.07) |  |
| **Knee ache** | | | | | |
| <60 | Reference | 1.57(1.36,1.81) | 0.34 | 1.05(1.04,1.06) | 0.69 |
| ≥60 | Reference | 1.55(1.31,1.83) |  | 1.04(1.03,1.06) |  |
| **Ankle ache** | | | | | |
| <60 | Reference | 1.60(1.31,1.96) | 0.31 | 1.06(1.04,1.07) | 0.64 |
| ≥60 | Reference | 2.00(1.59,2.51) |  | 1.06(1.04,1.08) |  |
| **Toe ache** | | | | | |
| <60 | Reference | 1.41(1.08,1.83) | 0.08 | 1.05(1.03,1.07) | 0.51 |
| ≥60 | Reference | 1.99(1.54,2.58) |  | 1.06(1.04,1.08) |  |
| **Neck ache** | | | | | |
| <60 | Reference | 1.42(1.19,1.70) | 0.09 | 1.04(1.03,1.06) | 0.34 |
| ≥60 | Reference | 1.95(1.57,2.43) |  | 1.06(1.04,1.08) |  |
| Adjusted for age, sex, WC, BMI, DBP, SBP, HDL-c, LDL-c, TC, TG, Cr, HbA1c, marital status, smoking, alcohol consumption status, history of diabetes, history of hypertension, history of stroke, and history of heart disease.  CESD, Center for Epidemiological Studies Depression Scale; HDL-c, glucose index; BMI, body mass index; WC, waist circumference; DBP, diastolic blood pressure; SBP, systolic blood pressure; LDL-c, low-density lipoprotein cholesterol; TG, triglyceride; Cr, creatinine. | | | | | |

| **Table S2 Hazard ratios and 95% CIs for the association of depression and CESD scores and pain at various sites throughout the body were calculated using gender as a subgroup** | | | | | |
| --- | --- | --- | --- | --- | --- |
| **Variables** | **Non-depressed** | **Depressed** | **P for interaction(Depressed)** | **Per SD** | **P for interaction(CESD)** |
| **Headache** | | | | | |
| Male | Reference | 1.91(1.55,2.36) | 0.02 | 1.08(1.06,1.09) | 0.24 |
| Female | Reference | 1.68(1.46,1.93) |  | 1.05(1.04,1.06) |  |
| **Shoulder ache** | | | | | |
| Male | Reference | 1.68(1.38,2.05) | 0.37 | 1.05(1.04,1.07) | 0.37 |
| Female | Reference | 1.46(1.27,1.68) |  | 1.04(1.03,1.05) |  |
| **Arm ache** | | | | | |
| Male | Reference | 1.83(1.47,2.28) | 0.23 | 1.06(1.04,1.08) | 0.16 |
| Female | Reference | 1.56(1.34,1.81) |  | 1.05(1.03,1.06) |  |
| **Wrist ache** | | | | | |
| Male | Reference | 2.01(1.53,2.64) | 0.18 | 1.07(1.04,1.09) | 0.28 |
| Female | Reference | 1.62(1.35,1.96) |  | 1.05(1.04,1.07) |  |
| **Finger ache** | | | | | |
| Male | Reference | 1.80(1.36,2.37) | 0.58 | 1.07(1.04,1.09) | 0.96 |
| Female | Reference | 1.92(1.63,2.27) |  | 1.06(1.05,1.08) |  |
| **Chest ache** | | | | | |
| Male | Reference | 2.20(1.68,2.89) | 0.41 | 1.09(1.07,1.11) | 0.07 |
| Female | Reference | 1.95(1.58,2.41) |  | 1.06(1.05,1.08) |  |
| **Stomach ache** | | | | | |
| Male | Reference | 2.01(1.57,2.59) | 0.69 | 1.07(1.05,1.09) | 0.64 |
| Female | Reference | 1.89(1.59,2.24) |  | 1.06(1.05,1.08) |  |
| **Back ache** | | | | | |
| Male | Reference | 1.95(1.55,2.45) | 0.39 | 1.07(1.05,1.09) | 0.09 |
| Female | Reference | 1.63(1.39,1.92) |  | 1.05(1.03,1.06) |  |
| **Lower back ache** | | | | | |
| Male | Reference | 1.66(1.41,1.95) | 0.30 | 1.06(1.04,1.07) | 0.07 |
| Female | Reference | 1.51(1.33,1.70) |  | 1.04(1.03,1.05) |  |
| **Hip ache** | | | | | |
| Male | Reference | 1.96(1.44,2.66) | 0.36 | 1.08(1.05,1.10) | 0.20 |
| Female | Reference | 1.65(1.34,2.03) |  | 1.06(1.04,1.08) |  |
| **Leg ache** | | | | | |
| Male | Reference | 1.63(1.35,1.96) | 0.74 | 1.06(1.04,1.07) | 0.87 |
| Female | Reference | 1.67(1.47,1.91) |  | 1.05(1.04,1.06) |  |
| **Knee ache** | | | | | |
| Male | Reference | 1.65(1.37,1.99) | 0.52 | 1.05(1.04,1.07) | 0.34 |
| Female | Reference | 1.52(1.33,1.73) |  | 1.04(1.03,1.05) |  |
| **Ankle ache** | | | | | |
| Male | Reference | 1.66(1.26,2.18) | 0.51 | 1.05(1.03,1.08) | 0.46 |
| Female | Reference | 1.79(1.50,2.15) |  | 1.06(1.04,1.07) |  |
| **Toe ache** | | | | | |
| Male | Reference | 1.74(1.26,2.42) | 0.78 | 1.06(1.03,1.09) | 0.66 |
| Female | Reference | 1.65(1.33,2.07) |  | 1.05(1.03,1.07) |  |
| **Neck ache** | | | | | |
| Male | Reference | 2.11(1.66,2.69) | 0.01 | 1.08(1.06,1.10) | <0.001 |
| Female | Reference | 1.44(1.22,1.70) |  | 1.04(1.03,1.05) |  |
| Adjusted for age, sex, WC, BMI, DBP, SBP, HDL-c, LDL-c, TC, TG, Cr, HbA1c, marital status, smoking, alcohol consumption status, history of diabetes, history of hypertension, history of stroke, and history of heart disease.  CESD, Center for Epidemiological Studies Depression Scale; HDL-c, glucose index; BMI, body mass index; WC, waist circumference; DBP, diastolic blood pressure; SBP, systolic blood pressure; LDL-c, low-density lipoprotein cholesterol; TG, triglyceride; Cr, creatinine. | | | | | |

| **Table S3 Hazard ratios and 95% CIs for the association of depression and CESD scores and pain at various sites throughout the body were calculated using smoking history as a subgroup** | | | | | |
| --- | --- | --- | --- | --- | --- |
| **Variables** | **Non-depressed** | **Depressed** | **P for interaction(Depressed)** | **Per SD** | **P for interaction(CESD)** |
| **Headache** | | | | | |
| No | Reference | 1.67(1.46,1.91) | 0.17 | 1.05(1.04,1.06) | 0.03 |
| Yes | Reference | 1.96(1.58,2.44) |  | 1.07(1.06,1.09) |  |
| **Shoulder ache** | | | | | |
| No | Reference | 1.50(1.31,1.71) | 0.84 | 1.04(1.03,1.05) | 0.85 |
| Yes | Reference | 1.60(1.30,1.96) |  | 1.05(1.03,1.06) |  |
| **Arm ache** | | | | | |
| No | Reference | 1.54(1.32,1.78) | 0.13 | 1.05(1.03,1.06) | 0.23 |
| Yes | Reference | 1.84(1.47,2.31) |  | 1.06(1.04,1.08) |  |
| **Wrist ache** | | | | | |
| No | Reference | 1.66(1.39,2.00) | 0.43 | 1.05(1.04,1.07) | 0.44 |
| Yes | Reference | 1.89(1.42,2.53) |  | 1.06(1.04,1.09) |  |
| **Finger ache** | | | | | |
| No | Reference | 1.84(1.56,2.17) | 0.66 | 1.06(1.05,1.07) | 0.58 |
| Yes | Reference | 1.98(1.49,2.61) |  | 1.07(1.04,1.09) |  |
| **Chest ache** | | | | | |
| No | Reference | 1.85(1.51,2.27) | 0.15 | 1.06(1.04,1.08) | 0.01 |
| Yes | Reference | 2.39(1.80,3.17) |  | 1.10(1.08,1.13) |  |
| **Stomach ache** | | | | | |
| No | Reference | 1.84(1.56,2.18) | 0.41 | 1.06(1.05,1.07) | 0.41 |
| Yes | Reference | 2.15(1.66,2.79) |  | 1.07(1.05,1.10) |  |
| **Back ache** | | | | | |
| No | Reference | 1.67(1.43,1.95) | 0.48 | 1.05(1.04,1.06) | 0.16 |
| Yes | Reference | 1.88(1.48,2.39) |  | 1.07(1.05,1.09) |  |
| **Lower back ache** | | | | | |
| No | Reference | 1.54(1.37,1.73) | 0.58 | 1.04(1.03,1.05) | 0.34 |
| Yes | Reference | 1.61(1.35,1.91) |  | 1.05(1.04,1.07) |  |
| **Hip ache** | | | | | |
| No | Reference | 1.60(1.30,1.97) | 0.19 | 1.06(1.04,1.07) | 0.05 |
| Yes | Reference | 2.10(1.54,2.87) |  | 1.09(1.06,1.11) |  |
| **Leg ache** | | | | | |
| No | Reference | 1.66(1.46,1.88) | 0.87 | 1.05(1.04,1.06) | 0.97 |
| Yes | Reference | 1.66(1.36,2.02) |  | 1.06(1.04,1.07) |  |
| **Knee ache** | | | | | |
| No | Reference | 1.54(1.35,1.75) | 0.56 | 1.05(1.03,1.06) | 0.67 |
| Yes | Reference | 1.62(1.33,1.97) |  | 1.05(1.03,1.07) |  |
| **Ankle ache** | | | | | |
| No | Reference | 1.81(1.52,2.17) | 0.53 | 1.06(1.05,1.08) | 0.64 |
| Yes | Reference | 1.64(1.23,2.17) |  | 1.05(1.03,1.08) |  |
| **Toe ache** | | | | | |
| No | Reference | 1.58(1.27,1.97) | 0.17 | 1.05(1.03,1.07) | 0.24 |
| Yes | Reference | 2.02(1.45,2.81) |  | 1.07(1.04,1.10) |  |
| **Neck ache** | | | | | |
| No | Reference | 1.50(1.27,1.76) | 0.11 | 1.04(1.03,1.05) | 0.05 |
| Yes | Reference | 2.00(1.54,2.58) |  | 1.07(1.05,1.09) |  |
| Adjusted for age, sex, WC, BMI, DBP, SBP, HDL-c, LDL-c, TC, TG, Cr, HbA1c, marital status, smoking, alcohol consumption status, history of diabetes, history of hypertension, history of stroke, and history of heart disease.  CESD, Center for Epidemiological Studies Depression Scale; HDL-c, glucose index; BMI, body mass index; WC, waist circumference; DBP, diastolic blood pressure; SBP, systolic blood pressure; LDL-c, low-density lipoprotein cholesterol; TG, triglyceride; Cr, creatinine. | | | | | |

| **Table S4 Hazard ratios and 95% CIs for the association of depression and CESD scores and pain at various sites throughout the body were calculated using drinking history as a subgroup** | | | | | |
| --- | --- | --- | --- | --- | --- |
| **Variables** | **Non-depressed** | **Depressed** | **P for interaction(Depressed)** | **Per SD** | **P for interaction(CESD)** |
| **Headache** | | | | | |
| No | Reference | 1.65(1.43,1.89) | 0.04 | 1.05(1.04,1.06) | 0.04 |
| Yes | Reference | 2.02(1.65,2.48) |  | 1.07(1.05,1.09) |  |
| **Shoulder ache** | | | | | |
| No | Reference | 1.55(1.35,1.78) | 0.81 | 1.05(1.04,1.06) | 0.53 |
| Yes | Reference | 1.50(1.23,1.83) |  | 1.04(1.02,1.06) |  |
| **Arm ache** | | | | | |
| No | Reference | 1.62(1.39,1.88) | 0.77 | 1.05(1.04,1.06) | 0.93 |
| Yes | Reference | 1.71(1.37,2.14) |  | 1.05(1.04,1.07) |  |
| **Wrist ache** | | | | | |
| No | Reference | 1.69(1.40,2.03) | 0.65 | 1.06(1.04,1.07) | 0.98 |
| Yes | Reference | 1.89(1.44,2.47) |  | 1.06(1.04,1.09) |  |
| **Finger ache** | | | | | |
| No | Reference | 1.86(1.58,2.20) | 0.69 | 1.06(1.05,1.08) | 0.98 |
| Yes | Reference | 1.91(1.47,2.49) |  | 1.06(1.04,1.08) |  |
| **Chest ache** | | | | | |
| No | Reference | 1.91(1.56,2.35) | 0.52 | 1.07(1.05,1.08) | 0.28 |
| Yes | Reference | 2.29(1.73,3.03) |  | 1.09(1.07,1.12) |  |
| **Stomach ache** | | | | | |
| No | Reference | 1.86(1.57,2.21) | 0.67 | 1.06(1.05,1.08) | 0.84 |
| Yes | Reference | 2.14(1.66,2.77) |  | 1.07(1.05,1.09) |  |
| **Back ache** | | | | | |
| No | Reference | 1.73(1.48,2.03) | 0.83 | 1.05(1.04,1.07) | 0.70 |
| Yes | Reference | 1.75(1.39,2.20) |  | 1.06(1.03,1.08) |  |
| **Lower back ache** | | | | | |
| No | Reference | 1.58(1.40,1.78) | 0.90 | 1.05(1.04,1.06) | 0.85 |
| Yes | Reference | 1.56(1.32,1.84) |  | 1.05(1.03,1.06) |  |
| **Hip ache** | | | | | |
| No | Reference | 1.69(1.37,2.09) | 0.54 | 1.06(1.04,1.08) | 0.42 |
| Yes | Reference | 1.87(1.38,2.55) |  | 1.07(1.04,1.10) |  |
| **Leg ache** | | | | | |
| No | Reference | 1.67(1.47,1.90) | 0.56 | 1.05(1.04,1.06) | 0.69 |
| Yes | Reference | 1.61(1.33,1.96) |  | 1.05(1.04,1.07) |  |
| **Knee ache** | | | | | |
| No | Reference | 1.49(1.31,1.70) | 0.24 | 1.04(1.03,1.05) | 0.26 |
| Yes | Reference | 1.77(1.47,2.15) |  | 1.06(1.04,1.08) |  |
| **Ankle ache** | | | | | |
| No | Reference | 1.80(1.50,2.15) | 0.72 | 1.06(1.04,1.07) | 0.90 |
| Yes | Reference | 1.68(1.28,2.20) |  | 1.06(1.03,1.08) |  |
| **Toe ache** | | | | | |
| No | Reference | 1.65(1.32,2.06) | 0.70 | 1.05(1.04,1.07) | 0.95 |
| Yes | Reference | 1.82(1.32,2.53) |  | 1.06(1.03,1.09) |  |
| **Neck ache** | | | | | |
| No | Reference | 1.58(1.34,1.86) | 0.67 | 1.05(1.03,1.06) | 0.42 |
| Yes | Reference | 1.70(1.33,2.18) |  | 1.06(1.04,1.08) |  |
| Adjusted for age, sex, WC, BMI, DBP, SBP, HDL-c, LDL-c, TC, TG, Cr, HbA1c, marital status, smoking, alcohol consumption status, history of diabetes, history of hypertension, history of stroke, and history of heart disease.  CESD, Center for Epidemiological Studies Depression Scale; HDL-c, glucose index; BMI, body mass index; WC, waist circumference; DBP, diastolic blood pressure; SBP, systolic blood pressure; LDL-c, low-density lipoprotein cholesterol; TG, triglyceride; Cr, creatinine. | | | | | |
